# Control of *Phlebotomus argentipes* (Diptera: Psychodidae) sand fly in Bangladesh: A cluster randomized controlled trial

**DOI:** 10.1371/journal.pntd.0005890

**Published:** 2017-09-05

**Authors:** Rajib Chowdhury, Shyla Faria, M. Mamun Huda, Vashkar Chowdhury, Narayan Prosad Maheswary, Dinesh Mondal, Shireen Akhter, Sakila Akter, Rajaul Karim Khan, Shah Golam Nabi, Axel Kroeger, Daniel Argaw, Jorge Alvar, Aditya Prasad Dash, Qamar Banu

**Affiliations:** 1 National Institute of Preventive and Social Medicine (NIPSOM), Mohakhali, Dhaka, Bangladesh; 2 International Centre for Diarrhoea Disease Research, Bangladesh (icddr,b), Dhaka, Bangladesh; 3 Department of Statistics, Dhaka College, Dhaka, Bangladesh; 4 Directorate General of Health Services (DGHS), Mohakhali, Dhaka, Bangladesh; 5 Mugda Medical College, Dhaka, Bangladesh; 6 Special Programme for Research and Training in Tropical Diseases, World Health Organization, Geneva, Switzerland; 7 University of Freiburg, Centre for Medicine and Society/Anthropology, Freiburg, Germany; 8 World Health Organization (WHO), Geneva, Switzerland; 9 Drugs for Neglected Diseases initiative (DNDi), Geneva, Switzerland; 10 Central University of Tamil Nadu, Thiruvarur, India; 11 Asian University for Women, Chittatong, Bangladesh; National Institute of Allergy and Infectious Diseases, UNITED STATES

## Abstract

**Background:**

A number of studies on visceral leishmaniasis (VL) vector control have been conducted during the past decade, sometimes came to very different conclusion. The present study on a large sample investigated different options which are partially unexplored including: (1) indoor residual spraying (IRS) with alpha cypermethrin 5WP; (2) long lasting insecticide impregnated bed-net (LLIN); (3) impregnation of local bed-nets with slow release insecticide K-O TAB 1-2-3 (KOTAB); (4) insecticide spraying in potential breeding sites outside of house using chlorpyrifos 20EC (OUT) and different combinations of the above.

**Methods:**

The study was a cluster randomized controlled trial where 3089 houses from 11 villages were divided into 10 sections, each section with 6 clusters and each cluster having approximately 50 houses. Based on vector density (males plus females) during baseline survey, the 60 clusters were categorized into 3 groups: (1) high, (2) medium and (3) low. Each group had 20 clusters. From these three groups, 6 clusters (about 300 households) were randomly selected for each type of intervention and control arms. Vector density was measured before and 2, 4, 5, 7, 11, 14, 15, 18 and 22 months after intervention using CDC light traps. The impact of interventions was measured by using the difference-in-differences regression model.

**Results:**

A total of 17,434 sand flies were collected at baseline and during the surveys conducted over 9 months following the baseline measurements. At baseline, the average *P*. *argentipes* density per household was 10.6 (SD = 11.5) in the control arm and 7.3 (SD = 8.46) to 11.5 (SD = 20.2) in intervention arms. The intervention results presented as the range of percent reductions of sand flies (males plus females) and rate ratios in 9 measurements over 22 months. Among single type interventions, the effect of IRS with 2 rounds of spraying (applied by the research team) ranged from 13% to 75% reduction of *P*. *argentipes* density compared to the control arm (rate-ratio [RR] ranged from 0.25 to 0.87). LLINs caused a vector reduction of 9% to 78% (RR, 0.22 to 0.91). KOTAB reduced vectors by 4% to 73% (RR, 0.27 to 0.96). The combination of LLIN and OUT led to a vector reduction of 26% to 86% (RR, 0.14 to 0.74). The reduction for the combination of IRS and OUT was 8% to 88% (RR, 0.12 to 0.92). IRS and LLIN combined resulted in a vector reduction of 13% to 85% (RR, 0.15 to 0.77). The IRS and KOTAB combination reduced vector densities by 16% to 86% (RR, 0.14 to 0.84). Some intermediate measurements for KOTAB alone and for IRS plus LLIN; and IRS plus KOTAB were not statistically significant. The bioassays on sprayed surfaces or netting materials showed favourable results (>80% mortality) for 22 months (IRS tested for 12 months). In the KOTAB, a gradual decline was observed after 6 months.

**Conclusions:**

LLIN and OUT was the best combination to reduce VL vector densities for 22 months or longer. Operationally, this is much easier to apply than IRS. A cost analysis of the preferred tools will follow. The relationship between vector density (males plus females) and leishmaniasis incidence should be investigated, and this will require estimates of the Entomological Inoculation Rate.

## Author summary

Integrated vector management (IVM) is of the important elements in the Regional Visceral Leishmaniasis (VL) elimination strategy. VL supposed to be eliminated from the Region (Bangladesh, India and Nepal) by 2015 which is extended up to 2017. There are several factors are responsible for not achieving the elimination goal and IVM is one of them. In Bangladesh, currently VL vector control activity is confined to indoor residual spraying with deltamethrin. Therefore, it is crucial to identify alternative VL vector control method(s) to enhance VL elimination programme. In the present study we have investigated the efficacy of (1) indoor residual spraying (IRS) with alpha cypermethrin 5WP; (2) long lasting insecticide impregnated bed-net (LLIN); (3) impregnation of local bed-nets with slow release insecticide K-O TAB 1-2-3 (KOTAB); (4) insecticide spraying in potential breeding sites using chlorpyrifos 20EC (OUT) and different combinations of the above. Study findings showed that the combination of LLIN and OUT was the most effective method against VL vector and among single interventions, IRS with two rounds of spraying found efficacious. In addition to IRS, VL elimination programme should consider LLIN plus OUT for vector control in relation to IVM.

## Introduction

Visceral leishmaniasis (VL) [known as kala-azar in the Indian sub-continent] is a parasitic disease present in South-East Asia since before the early 1800’s [1]. VL appears to have spread along the Ganges and the Brahmaputra rivers, the major transport routs of Bengal and Bangladesh. In this area, VL was first described in 1824 in the Jessore district where about 75,000 people died [2]. An intensive control programme aimed at the eradication of malaria was mounted in the late 1950s and early 1960s throughout the South Asian sub-continent with the main effort based on indoor residual spraying (IRS) of DDT (Dichlorodiphenyltrichloroethane). During the malaria eradication programme the incidence of VL dropped dramatically as a collateral benefit with DDT spraying [3]. However, within a few years after the end of the Malaria eradication effort, VL returned to Bihar and Bengal on both sides of the borders of India and Bangladesh [4].

In Bangladesh, five districts, Sirajgang, Pabna, Mymensingh, Rajshahi and Tangail were more affected following the end of the malaria eradication programme to 1980s [5]. These districts continue to have the highest number of cases along with other districts reporting few cases. The Malaria and Vector Borne Disease Control Unit, Directorate General of Health Service (DGHS), Government of Bangladesh has reported 109,226 VL cases including 329 VL related deaths from 1994 to 2013 [6]. Mymensingh district alone contributed about 50% of total reported cases and the highest number of cases was reported from the Fulbaria upazila (sub-district) with several other upazilas in Mymensingh reporting cases [6]. To combat VL in the Indian Sub-continent, a common platform was developed through signing a memorandum of understanding (MoU) by the health ministers from the three affected countries (Bangladesh, India and Nepal) in 2005 [7]. In the MoU, a target was set to reduce the VL incidence to less than 1 case per 10,000 population at the sub-district (upazila in Bangladesh) level by 2015 [7]. This MoU has been extended up to 2017 with inclusion of Bhutan and Thailand in the group [8]. Integrated vector management is one of the most important pillars in the elimination strategy, however virtually no vector control activities were under taken in Bangladesh for VL vector control until 2010 [6,9].

Since 2011, the National Kala-azar Elimination Programme (NKEP) in Bangladesh conducted IRS for vector control using deltamethrin 5 WP in the affected communities. In addition to IRS, two commercially manufactured long lasting impregnated bed-nets (LLIN) were given to each patient who was treated in the government hospitals for the last three years (2011 to 2013). The IRS (using deltamethrin 5WP) and LLIN were associated to decrease the level of the *Phlebotomus argentipes* sand fly by 70–80% in Bangladesh [10]. In India and Nepal, deficiencies in the quality of IRS was observed when carried out by the national programme [11]. Mosquito nets impregnated with a slow release insecticide (K-O TAB 1-2-3; deltamethrin with a binder) resulted in a 65% reduction in sand fly levels in Bangladesh [12]. None of the studies however determined the effect of both LLIN and insecticide treated local nets in a single study. VL vectors usually breed in the shady places with loose soil where enough moisture is available around the houses [13]. There is lack of evidence in Bangladesh for controlling immature stages of *P*. *argentipes* sand fly by applying insecticide spraying on their breeding habitat around the houses. Hypothetically, it is believed that the combined application of two vector control methods will increase the effect on VL vectors but currently there is no evidence for this in South-East Asia.

The present study on single and combined vector control interventions was conducted to assist the NKEP by identifying suitable VL vector control method(s) to achieve the elimination target within the set time frame and maintain a low vector density during the maintenance phase of the VL elimination programme.

## Methods

### Study site and population

#### Selection of study area

Fulbaria upazila (sub-district) of Mymensingh district was selected as the study area since the highest number of visceral leishmaniasis (VL) cases has been reported from this upazila during the period of 2009 to 2011. A total of 2,845 cases were reported in three years, of which 781, 456 and 1608 cases were reported in 2009, 2010 and 2011 respectively [6]. From study villages (the names of villages and their respective unions are mentioned below in the ‘selection of study villages’), total 42 cases (6 in 2009; 17 in 2010 and 19 in 2011) were reported. Fulbaria is 111 kilometres away from the capital city Dhaka and 23 km from the Mymensingh district headquarter. Fulbaria upazila has 13 unions (lowest administrative unit) including a Paurashava and has 116 villages. The upazila occupies an area of 398.70 square kilometer (KM) including 14.76 square KM forest area. It is located between 24°23' and 24°44' north latitudes and between 90°08' and 90°28' east longitudes [14]. The maximum elevation above sea level of Mymensingh district is 52 feet (15.85 meter) [15]. During the last census in 2011, in Fulbaria upazila, total population was 448,467 of which 222,901 male and 225,566 female with 1.23 annual growth rate. The population density is 1125 per square km. Total HH is 101,189 and average family size is 4.43. The literacy rate for both sexes is 42.3% of which 43.6% for male and female is 41.1% [14]. In Bangladesh, monsoon starts in June and stays up to October [16] and the dry season starts in mid-November and continues to early-March [17]. In Fulbaria upazila have non-calcareous dark grey floodplain and red-brown terrace soils [18]. The pH value of Mymensingh district lies between 5.5 and 6 [16].

### Sample size calculation

The sample size estimation was based on the vector densities (female and male *P*.*argentipes* sand fly counts per household), variations and distributions documented in previous entomological studies and sand fly reduction rates in similar intervention studies performed in Venezuela; Bangladesh, India and Nepal [19,20]. Based on our previous experience, male:female ratio was always about 50:50 throughout the year [10,20], and so we assume that joint counts will not affect the outcome of the analysis, but will produce more accurate estimate through regression model. We assumed that the distribution of sand fly counts would follow a negative binomial distribution with a dispersion coefficient of *k* = 0.05 and an intra-cluster coefficient of 0.03, a reduction from 20 to 5 vectors per trap/ night, and an average of 50 households per cluster. The minimum sample size was found to be 6 clusters per intervention arm, with a total of 60 clusters in the study to achieve 80% power and a significance level of 5%.

### Selection of study villages

**Fig 1:**
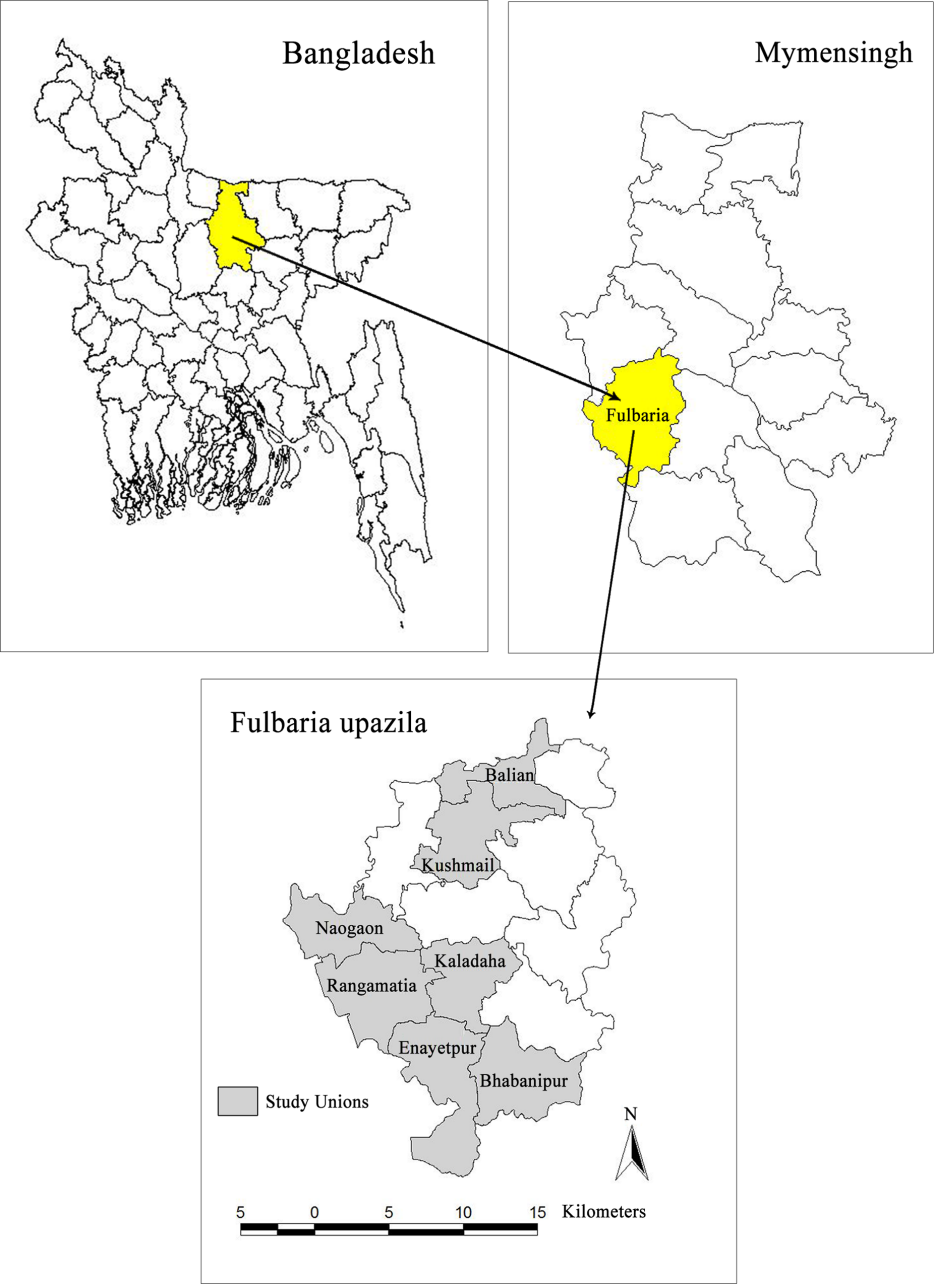
Study areas.

VL surveillance data for three years (2009–2011) was collected from Upazila Health Complex (UHC), Fulbaria. Based on the passive surveillance reports, endemic villages were identified (containing 300 to 600 households [HH]) and were selected where the national programme was not conducting routine vector control activities. The following villages were selected (Fig 1): Mahespur in Bhabanipur union (311 HHs); Mandolbari and Chalkgarbajail in Balian union (319 HHs); Patira in Kaladah union (323 HHs); Hurbari in Kaladah union (297 + 312 = 609 HHs); Dulma in Enayetpur union (309 HHs); Kathgarh in Naogaon union (307 HHs); Bisania and Natuapara in Kushmail union (300 HHs); Anuhadi in Rangamatia union (322 HHs); and Haripur in Rangamatia union (302 HHs), in total 3079 HHs). We had no indication or evidence that the study villages were atypical. The study was conducted from September 2012 to October 2014.

### Selection of study “sections” within villages

**Fig 2:**
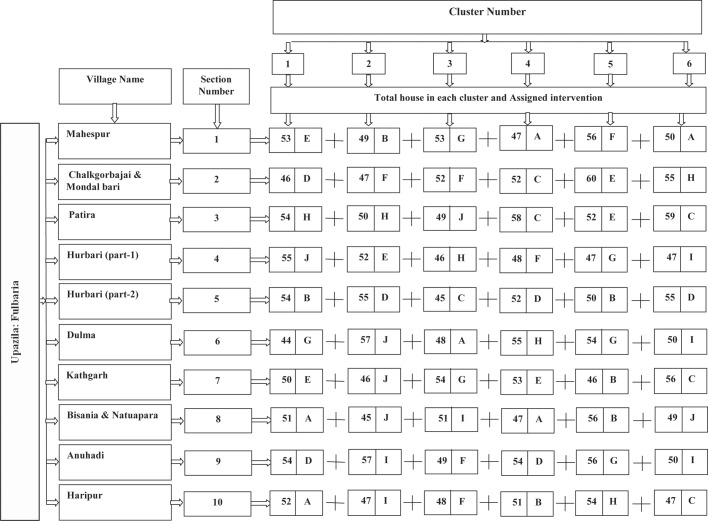
Study design. [Note: Interventions: IRS = A; LLIN = B; Local bednet impregnated with KO TAB 1-2-3 = C; Possible breeding places (outside of home) sprayed with Clorophyrephos = D; A+B = E; A+C = F; B+D = G; C+D = H; A+D = I and J = Control (no intervention)]

Based on their high endemicity levels, 11 villages were selected from seven unions. Eleven villages were divided into 10 sections with a minimum of 300 HHs each (Fig 2).

### Selection of clusters within sections

Each section with at least 300 HHs was divided into six clusters, each with about 50 HHs. In each study cluster, all HHs were numbered using enamel paint on the front door. There was a minimum of 50 meters distance between two clusters to avoid cross-contamination of the interventions. The total number of 60 clusters (10 sections x 6 clusters each) was assigned for implementation of interventions (Fig 2).

### Selection of HHs within clusters for the entomological surveys

Five HHs from each cluster (5 HHs x 60 clusters = 300 HHs) were selected by simple random sampling for measuring sand fly densities at 3 weeks before intervention (baseline survey) and at 2, 4, 5, 7, 11, 14, 15, 18 and 22 months after intervention (follow-up surveys).

In order to achieve homogenous and comparable groups of clusters, the following approach was undertaken. Based on vector (*P*. *argentipes*) density during the baseline collection, the 60 study clusters were stratified into 3 groups: (i) high [range of *P*. *argentipes*– 59 to 143], (ii) medium [range of *P*. *argentipes*– 31 to 57] and (iii) low [range of *P*. *argentipes*– 1 to 30] vector density. Each group (vector density stratum) had 20 clusters. From these 60 clusters (20 clusters in each group), the different intervention and control arms were randomly selected and each arm had 6 clusters [about 300 HHs each] (Fig 2).

### Household interviews

After the formation of the clusters, all HH heads were interviewed by trained field staff (Research Assistants [RA]) using a structured questionnaire to record the HH information, socioeconomic characteristics and some epidemiological information.

### Description of intervention arms

1. ***Indoor residual spraying (IRS)*:** The inside of the all house walls were sprayed with Alpha Cypermethrin 5 WP (manufactured by Tagros Chemical India Ltd.) up to 6 feet (183 cm).
2. ***Long lasting insecticidal net (LLIN)*:** Long lasting insecticide synergist combination bed net PermaNet 3.0 manufactured by Vestergaard Frandsen Private Ltd were used and had the following characteristics; size: 190 × 180 × 150 cm; Color: white side and blue rooftop; mesh size: minimum 20 holes/cm^2^ and 75 denier with 70 cm lower border. Pyrethroid deltamethrin and the synergist piperonyl butoxide [PBO] were incorporated into monofilament polyethylene yarn of 100 deniers at the target dosage of 4.0 g AI/kg and 25 g AI/kg of netting material respectively on the roof of the net by the manufacturer. The side panels of PermaNet 3.0 are made of multi-filament polyester fibres, treated with deltamethrin in a resin coating. The side netting has two parts: a strengthened lower part, so-called border (70 cm) by using 75 ± 5% denier yarn (weight 40 ± 10% g/m^2^) and a side panel made of 75 ± 5% denier (weight of 30 ± 10% g/m^2^). The target dosage of deltamethrin in the side panels is 2.8 g AI/kg of netting material, i.e. 115 mg AI/m^2^ of the border and 85 mg AI/m^2^ of the remaining of the side panels. PBO is generally used in commercial aerosols for potentiating pyrethroid activity against P450s and esterases in flying or domestic insect pests [21]. The LLINs were donated to each HH of selected clusters according to their sleeping arrangement.
3. ***Slow release insecticide treated bed nets (KOTAB)*:** In the selected HHs of study villages, bed nets were impregnated with slow release insecticide (deltamethrin) tablet K-O TAB 1-2-3; manufactured by Bayer Crop Science.
4. ***Insecticide spraying in the possible breeding places (OUT)*:** Possible sand fly breeding places around the houses of all HHs in the selected clusters were sprayed with Chlorpyrifos (Aciphos 20EC, manufactured by ACI Crop Care and Public Health). This insecticide is registered in Bangladesh for public health use [22] and World Health Organization Pesticide Evaluation Scheme (WHOPES) also approved it for control of mosquito larvae [23]. Outside of houses possible sand fly breeding places includes shady places with moisture and debris; cow dung pit (places of cow dung storage); feeding troughs of cattle (without shade); surroundings of tube well and latrines; beneath of human dwelling and cattle shade (outside of house); etc. [13].
5. ***Combined intervention*:** The following combinations were implemented in the selected HHs: (i) IRS and LLIN, (ii) IRS and KOTAB, (iii) IRS and OUT, (iv) LLIN and OUT and (v) KOTAB and OUT.
6. ***Control (no intervention)*:** No intervention was undertaken during the study period. After completion of the study, each HH in this arm received one or more LLINs.

### Implementation of interventions

a ***Training of community volunteer and Spraymen***
  We selected 15 motivated and energetic volunteers from the study areas and most of them had a secondary school degree. They assisted with the bed net dipping programme in the community. Similarly, 15 spraymen (who have trained and worked with the national IRS programme) were selected from the study villages or nearby endemic communities. A two day training (in-house and practical) was provided to the community volunteers on bed net dipping procedures using manufacturer’s (Bayer Crop Science) guideline by the investigators. Similarly, investigators provided two day training (in-house and practical) to spraymen on the proper spraying techniques (following ‘Monitoring and evaluation tool kit for indoor residual spraying’ [24]). No rewards or incentives were provided to anyone during implementation of interventions.
b ***IRS implementation***
  To prepare houses (mud wall plastering or cleaning of pacca/tin wall) for IRS, all HHs were visited two weeks before IRS by field staff (RA) and informed about the procedures and the insecticides. All HHs were visited again by field staff one week before spraying to ensure HHs were prepared. Spraying was carried out under the supervision of one investigator.
  IRS was conducted twice during the study period: (i) December 2012 and (ii) September 2013. Eight liter Hudson Expert pumps (H.D. Hudson Manufacturing Company, Chicago, IL) were used for spraying; 100 gm insecticide sachets were mixed with 8 liters of water. This quantity covered one house.
c ***LLIN distribution***
  All HHs were visited in the selected study clusters to observe peoples`sleeping arrangement for identifying bed net requirements prior to distributing the nets to all HHs in December 2012. Each HH received an average 1.88 pieces of LLIN in the selected clusters. On the day of distribution, all HHs were instructed to use the nets every day during sleeping at night.
d ***Bed net dipping with K-O TAB 1-2-3 (KOTAB)***
  Field staff visited selected HHs three to four weeks before the impregnation programme to provide information about the bed net dipping programme with slow release insecticide (K-O TAB 1-2-3). During the visit, HH residents were asked to wash their existing nets or to buy new nets (those HH without nets). HHs were also informed about the day and place of the dipping programme which was usually performed in the centre of the cluster. During the visit, field staff marked all existing nets by writing the HH number on them to avoid conflicts among the villagers and also to count the total number of nets available per HH. One week before the intervention, field staff visited all HHs to ensure that all available nets were cleaned properly and ready for dipping and that the new nets got a number. The dipping programme was organized in December 2012. In the selected clusters, an average 1.85 pieces of nets were dipped in each HH.
e ***Outdoor spraying (OUT)***
  One week before spraying, field staff visited all selected HHs and informed the responsible family members about the schedule of spraying in the possible sand fly breeding places around the houses with Chlorpyrifos 20EC. HHs were asked not to remove their domestic animals on the day of spraying. Spraying was carried out by spraymen under the supervision of an investigator. Spraying was conducted twice during the study period: (i) December 2012 and (ii) September 2013. A Hudson expert pump (8 liters quantity) was used for spraying and 150 ml insecticide mixed with eight liters of water. An average of eight liters of insecticide mixed solution was used to cover breeding sites around each house.
f ***Combined intervention***
  Clusters assigned for combined interventions followed the same individual procedures outlined above.
g ***Supervision and monitoring***
  Investigators have substantial experience in entomological activities so that they were able to ensure the quality of work and performance of spraymen and volunteers by visual observation of the daily activities having been carried out. Moreover, investigators always monitored the study activities during the implementation of interventions.

### Entomological measurements

a **Sand fly density measurement, preservation and identification**
  Sand flies were collected from 300 HHs (60 clusters of 10 sections) using CDC light traps for two consecutive nights and used single trap per night per household for all houses. Traps were set up in the suitable places inside houses (kept at a corner about 6 inches (15.24 cm) away from the wall and about 2 inches (5.08 cm) above the floor) at dusk and collected on the following dawn. Collected sand flies were preserved in absolute ethanol. Sand flies were identified in the field office by a trained Research Officer under the direct supervision of investigators using a phase contrast stereo binocular microscope following taxonomic key described by Lewis 1978 and 1982 [25,26]. The following schedule was followed; (1) baseline (before intervention) in October and November 2012; (2) first follow up in February 2013; (3) second follow up in April 2013; (4) third follow up in May 2013; (5) forth follow up in July 2013; (6) fifth follow up in November 2013; (7) sixth follow up in February 2014; (8) seventh follow up in March 2014; (9) eighth follow up in June 2014 and (10) Ninth follow up in October 2014.
b **Bioassays**
  (i) **Bioassay on sprayed surfaces (wall)**
    A bioassay test on walls was conducted in the 40 randomly selected houses of sprayed villages and 40 in the control houses of unsprayed villages following WHOPES guidelines at room temperature of 28° to 32°C and 75 to 85% relative humidity. In each house, 10 to 12 female sand flies (caught using manual aspirator from unsprayed villages in the same study area) were exposed for 30 minutes on each of the four walls, using standard WHO plastic cones [27]. Mortality of the test sand flies was recorded at 24 hours post-exposure [28]. Corrected mortality was calculated, using the formula of Abbot (1925) [29]: 100(Pi-C)/(100-C), where Pi and C are the percentage mortalities observed in the sprayed and unsprayed houses, respectively. The bioassays were performed two times in each test house: 2 and 5 months after IRS for each spraying round.
  (ii) **Bioassay on LLIN and KO TAB 123 impregnated nets**
    Cone bioassays were performed in the field laboratory for LLINs and K-O TAB 1-2-3 impregnated nets following a similar procedure as described above. Wild caught 10 to 12 female sand flies were exposed to the impregnated netting material for 3 minutes under standard WHO plastic cones. Five randomly selected spots were checked for each bed net (4 spots from 4 sides of the net and 1 spot on the top of the net). Mortality of sand flies after 24 hours was observed and recorded. Bioassays were carried out on a total of 80 randomly selected nets (LLIN-40 and K-O TAB 1-2-3 impregnated nets-40) and 40 pieces of untreated polyester nets as a control. Bioassays of K-O TAB 1-2-3 impregnated nets and control nets (un-impregnated nets) were carried out at 3, 6, 12 and 20 months after impregnation and at 6, 12 and 20 months for the LLIN intervention. Corrected mortality was calculated as described above.

### Data management and statistical analysis

A standard data entry interface was designed using Microsoft Office Access for entering the study data. Data were checked and cleaned before analysis. Descriptive analysis was performed to determine the nature of the data. The main analysis was based on *P*. *argentipes* sand fly counts per HH collected by CDC light traps over two nights. No zero count was removed. All CDC traps worked perfectly. The average sand fly density among different interventions and control groups at baseline as well as follow-up time points was determined. Mean *P*. *argentipes* sand fly count between control and intervention areas were compared using a non-parametric approach (Mann Whitney U test). It was found that the negative binomial distribution fitted the data and all analyses were performed under that assumption. A generalized estimating equation (GEE) modelling technique was used to adjust for data correlations due to the longitudinal/ repeated measurements in cluster sampling. An interaction term for the intervention arm at follow-up was included in this model in order to estimate the effect of the intervention.

Technically, the regression model had the following structure:

Count=Intercept+a*Treatment+b*Time+c*Interaction+error

where treatment is one if it is the intervention and zero if it is the control; where time is one if follow up and zero if baseline; and where interaction is one if the intervention group at follow up. Intervention effect was measured using incidence rate ratio (IRR) of *P*. *argentines* sand fly count generated from the exponent of c-coefficient in the model. In the tables, IRR represented as the rate ratio (RR) and its p-value are given. Significances stated at 5% level and 95% confidence intervals are given. The main outcome variable was “*P*. *argentipes* sand flies per household” at before and 2, 4, 5, 7, 11, 14, 15, 18 and 22 months after the intervention. The following variables were controlled for in the full model: cattle shed, family members sleep in the bed room, number of bed nets in the house, type of house wall, presence of crack in wall and socio economic status. Economic status of the household was measured through the HH asset index. Household asset index was generated by the principal component method in factor analysis using the following variables: electricity, radio, television, mattress, bed net, motor cycle, bicycle, van, power tiller, shallow machine, chair/table, mobile phone, clock, sewing machine, and fishery. We categorized the index as low (score less than 33^th^ percentile), medium (score between 33^th^ to 66^th^ percentile) and high (score greater than 66^th^ percentile). We did not perform any sensitivity analysis as the study objective was to compare the efficacy of different vector control interventions against control arm. All analyses were performed by using STATA 10.1.

### Quality control

Investigators and external experts conducted all training activities to ensure the quality of the training. All study activities were monitored by the investigators to maintain the quality.

### Ethical consideration

The study was approved by Bangladesh Medical Research Council (BMRC). Written informed voluntary consent was obtained from the HH heads/responsible family members before conducting any study related activities. All control (no intervention) arm HHs were donated one LLIN per family after completing the study.

## Results

### Characteristics of the study population

The study was conducted in 3079 HHs with a population of 13,406 inhabitants in 10 sections of 60 clusters (Fig 2). Nine types of interventions were tested and one control arm. Within the study population, 49.4% were female and 39.6% were below 17 years of age (Table 1). About 5% and 0.6% of the total population had a past history of VL and PKDL respectively. The proportion of VL and PKDL varied from 3.6% to 5.8% and 0.1% to 1.1% respectively among the different study arms (Table 1). About 60% (1846/3079) of HHs had a cattle shed. The percentage of the population with cattle sheds varied from 53.5% (161/301) to 69.5% (214/308) among the different study arms. Almost all HHs had a non-impregnated bed net (99.1%) with an average of 2.33 per HH (SD = 1.35). About 30% of houses had precarious walls and about 15.0% had cracks in their walls. The study HHs were almost equally distributed among low (33.4%) medium (30.7%) and high (35.6%) asset index groups (Table 1).

**Table 1:** Study profile in Fulbaria, Mymensingh, Bangladesh.

Statement	Interventions										Overall
	IRS	LLIN	KOTAB	OUT	IRS+LLIN	IRS+KOTAB	IRS+OUT	LLIN+OUT	KOTAB+OUT	Control	
Number of cluster	6	6	6	6	6	6	6	6	6	6	60
Number of households, M	295	306	317	316	320	300	302	308	314	301	3079
Number of total population, N	1341	1398	1336	1370	1401	1236	1363	1326	1325	1310	13,406
Female, % N	49.2	50.4	48.7	50.6	49.1	48.4	49.6	50.1	49.1	48.2	49.4
Child (<17 years) % N	39.1	40.0	38.1	37.7	40.3	39.2	42.6	38.5	38.8	41.6	39.6
Past history of VL % N	4.1	4.2	4.5	5.8	5.7	3.6	5.0	4.1	4.2	3.7	4.5
Past history of PKDL % N	0.6	0.8	0.1	0.1	1.4	0.6	0.4	0.9	0.3	1.1	0.6
Cattle shed; % M	56.6	62.7	63.7	62.0	55.0	59.0	59.3	69.5	58.0	53.5	60.0
Bed-net available in house, % M	98.6	100	100	99.1	99.4	99.0	99.0	100	98.4	97.7	99.1
Number of bed-net in house, mean (SD)	2.08 (1.01)	3.48 (1.81)	1.96 (0.85)	1.96 (0.96)	2.79 (1.6)	1.94 (0.91)	1.96 (1.01)	3.17 (1.68)	1.92 (0.85)	2.02 (1.09)	2.33 (1.35)
Family member sleep in the main bed room, mean (SD)	3.98 (1.62)	3.87 (1.5)	3.95 (1.67)	3.71 (1.51)	3.82 (1.51)	3.84 (1.37)	4.06 (1.61)	3.73 (1.43)	3.56 (1.5)	3.89 (1.54)	3.84 (1.53)
Precarious house wall, % M	21.7	29.4	33.1	19.0	34.4	18.0	25.2	42.5	43.6	30.6	29.8
Cracks in wall, % M	11.5	17.6	18.3	17.1	19.4	7.0	15.9	16.2	15.3	11.6	15.1
Socio economic status											
Low % M	53.6	36.6	26.8	39.2	18.1	38.0	29.1	34.1	23.2	37.2	33.4
Medium % M	25.4	33.0	36.3	31.6	35.3	27.7	29.5	30.8	31.2	24.9	30.7
High % M	21.0	30.4	36.9	29.1	46.6	34.3	41.4	35.1	45.5	37.9	35.9

Intervention: Indoor residual spraying (IRS), Long lasting insecticide impregnated bed-net (LLIN), Local nets impregnated with slow release insecticide tablets K-O TAB 1-2-3 (KOTAB), Possible sand fly breeding places around house sprayed with chlorpyrifos (OUT), control = no intervention provided

### Sand fly densities

A total 17,434 sand flies were collected during the entire study period including baseline and the 9 follow-up surveys (Table 2). Of all sand flies, 53.75% were *P*. *argentipes* and the remainder other species. Among *P*. *argentipes*, 4,443 (47.42%) were female including 16.01% gravid. At baseline (before implementation of intervention), 3,616 sand flies were captured of which 78.32% were *P*. *argentipes*. There were 83 (18.12%), 412 (36.52%), 1315 (42.57%), 1443 (58.52%), 383 (55.59%), 67 (35.26%), 331 (55.72%), 1419 (37.92%) and 1085 (74.21%) *P*. *argentipes* sand flies collected respectively in the first to ninth follow up measurements. There is no significant difference between male and female ratio of collected *P*. *argentipes* sand flies throughout the study period (S1 Table).

**Table 2:** Total number of sand flies collected in all study houses during study period by species, sex, gravidity, feeding status in Fulbaria, Mymensingh, Bangladesh.

Species	Gender	Status	Measurement/collection period [%]																
			Baseline	First follow up		Second follow up	Third follow up		Forth follow up	Fifth follow up		Sixth follow up		Seventh follow up		Eight follow up		Ninth follow up	Total
*Phlebotomus argentipes*																			
	Female	Total	1409[49.75]	28[33.73]		184[44.66]	622[47.30]		651[45.11]	190[49.61]		24[35.82]		168[50.76]		602[42.42]		565[52.07]	4443 (47.42)
		Gravid	489[17.27]	15[18.07]		87[21.12]	250 [19.01]		161[11.16]	60[15.67]		2[2.99]		49[14.80]		230[16.21]		157[14.47]	1500 [16.01]
		Fed	0 [0]	0 [0]		0 [0]	0 [0]		0 [0]	0 [0]		0 [0]		0 [0]		0 [0]		0 [0]	0
		Neither	920[32.49]	13[15.66]		97[23.54]	372[28.29]		490[33.96]	130[33.94]		22[32.84]		119[35.95]		372[26.22]		408[37.60]	2943 [31.41]
	Male		1423[50.25]	55[66.27]		228[55.34]	693 [52.70]		792[54.89]	193[50.39]		43[64.18]		163[49.24]		817[57.58]		520[47.93]	4927 (52.58)
	Total		2832 [78.32]	83[18.12]		412 [36.52]	1315 [42.57]		1443 [58.52]	383 [55.59]		67 [35.26]		331 [55.72]		1419 [37.92]		1085 [74.21]	9370 [53.75]
Other species			793	375	716		1774	1023		306	123		263		2323		377		8073
**Total**			**3616**	**458**		**1128**	**3089**		**2466**	**689**		**190**		**594**		**3742**		**1462**	**17434**

Follow up/measurement period: (1) baseline (before intervention) in October and November 2012; (2) first follow up in February 2013; (3) second follow up in April 2013; (4) third follow up in May 2013; (5) forth follow up in July 2013; (6) fifth follow up in November 2013; (7) sixth follow up in February 2014; (8) seventh follow up in March 2014; (9) eighth follow up in June 2014 and (10) Ninth follow up in October 2014

**Fig 3:**
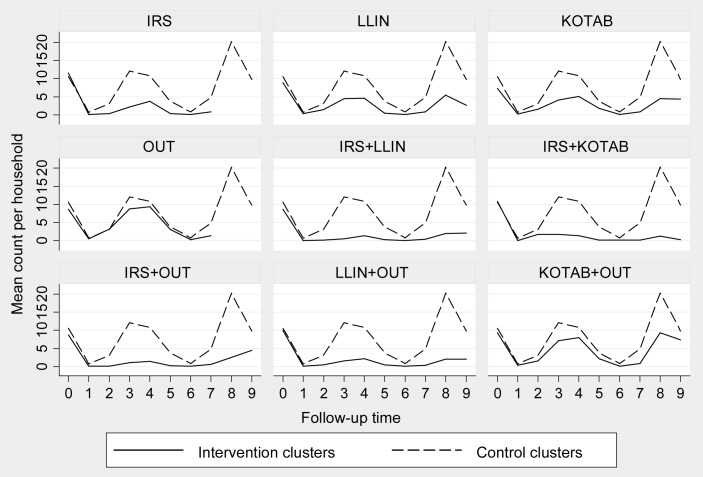
Distribution of *P*. *argentipes* sand fly over the study period in different interventions.

At baseline, the average *P*. *argentipes* density per household was 10.57 (SD = 11.51) in the control arm and 7.3 (SD = 8.46) to 11.53 (SD = 20.17) in the different intervention arms (Table 3). The difference of *P*. *argentipes* sand fly densities among intervention arms and control arm were not statistically significant at baseline except for the KOTAB intervention arm (p = 0.032). However, *P*. *argentipes* sand fly densities in most of the intervention arms were significantly lower than the control arm at different follow-up measurements except OUT (Table 3). Fig 3 shows that the mean *P*. *argentipes* sand fly density was always below the values in the control arm throughout all the measurements.

**Table 3:** Density of *P*. *argentipes* per household and their comparison between interventions and control arm at baseline and different follow-up time in Fulbaria, Mymensingh, Bangladesh.

	Total *P*. *argentipes* per household, Mean (95% CI) [P-value*]									
Survey/follow up	IRS	LLIN	KOTAB	OUT	IRS+LLIN	IRS+KOTAB	IRS+OUT	LLIN+OUT	KOTAB+OUT	Control
Baseline (Oct-Nov-12)	11.53(10.35, 12.81)[0.204]	8.8 (7.77, 9.93)[0.684]	7.3 (6.37, 8.33) [0.032]	8.7 (7.68, 9.82) [0.728]	8.63 (7.61, 9.75) [0.407]	10.83(9.69, 12.08)[0.495]	8.7 (7.68, 9.82) [0.207]	9.97 (8.87, 11.16)[0.238]	9.37 (8.30, 10.53)[0.157]	10.57 (9.44, 11.80) [–]
First (Feb-13)	0.17 (0.05, 0.39) [0.008]	0.37 (0.18, 0.66) [0.135]	0.2 (0.07, 0.44) [0.011]	0.53 (0.30, 0.87) [0.203]	0 (0.0,0.0) [0.000]	0.1 (0.02, 0.29)[0.001]	0.07 (0.01, 0.24)[0.001]	0.17 (0.05, 0.39) [0.013]	0.4 (0.21, 0.70) [0.140]	0.73 (0.46, 1.11) [–]
Second (Apr-13)	0.37 (0.18, 0.66) [0.000]	1.4 (1.01, 1.89)[0.003]	1.6 (1.18, 2.12) [0.017]	3.17 (2.56, 3.87) [0.782]	0.17 (0.05, 0.39) [0.000]	1.77 (1.32, 2.31) [0.000]	0.1 (0.02, 0.29) [0.000]	0.47 (0.26, 0.78) [0.000]	1.57 (1.15, 2.08) [0.001]	3.13 (2.53, 3.83) [–]
Third (May-13)	2.17 (1.67, 2.76) [0.000]	4.43 (3.71, 5.25) [0.072]	4.1 (3.41, 4.89) [0.012]	8.8 (7.77, 9.93) [0.212]	0.57 (0.33, 0.91) [0.000]	1.8 (1.35, 2.35) [0.000]	1.03 (0.70, 1.47) [0.000]	1.6 (1.18, 2.12)[0.000]	7.1 (6.18, 8.12) [0.051]	12.1 (10.89, 13.41) [–]
Fourth (Jul-13)	3.77 (3.10, 4.53) [0.000]	4.57 (3.83, 5.40) [0.010]	5.13 (4.35, 6.01)[0.001]	9.4 (8.33, 10.56)[0.609]	1.37 (0.98, 1.85) [0.000]	1.43 (1.04, 1.93) [0.000]	1.5 (1.09, 2.01) [0.000]	2.17 (1.67, 2.76) [0.000]	7.97 (6.99, 9.04) [0.039]	10.83 (9.69, 12.08) [–]
Fifth (Nov-13)	0.33 (0.16, 0.61) [0.000]	0.53 (0.30, 0.87) [0.000]	1.83 (1.38, 2.39) [0.002]	3.03 (2.44, 3.72) [0.125]	0.27 (0.11, 0.53) [0.000]	0.13 (0.04, 0.34) [0.000]	0.2 (0.07, 0.44) [0.000]	0.47 (0.26, 0.78) [0.000]	2.13 (1.64, 2.72) [0.002]	3.8 (3.13, 4.56) [–]
Sixth (Feb-14)	0.1 (0.02, 0.29) [0.001]	0.17 (0.05, 0.39) [0.008]	0.17 (0.05, 0.39) [0.008]	0.3 (0.14, 0.57) [0.016]	0.07 (0.01, 0.24)[0.000]	0.17 (0.05, 0.39) [0.008]	0.07 (0.01, 0.24) [0.000]	0.07 (0.01, 0.24) [0.000]	0.13 (0.04, 0.34) [0.002]	0.83 (0.54, 1.23) [–]
Seventh (Mar 14)	0.83 (0.54 1.23) [0.000]	0.9 (0.59, 1.31) [0.000]	0.8 (0.51, 1.19) [0.000]	1.37 (0.98, 1.85)[0.000]	0.4 (0.21, 0.70) [0.000]	0.13 (0.04, 0.34) [0.000]	0.57 (0.33, 0.91) [0.000]	0.3 (0.14, 0.57) [0.000]	0.8 (0.51, 1.19) [0.000]	4.87 (4.11, 5.72) [–]
Eight (Jun-14)	Not done	5.47 (4.66, 6.37) [0.030]	4.43 (3.71, 5.25) [0.005]	Not done	2.03 (1.56, 2.61) [0.000]	1.23 (0.87, 1.70) [0.000]	2.5 (1.97, 3.13) [0.000]	2.1 (1.61, 2.69) [0.000]	9.27 (8.21, 10.42) [0.050]	20.27 (18.69, 21.94) [–]
Ninth (Oct-14)	Not done	2.67 (2.11, 3.32) [0.001]	4.33 (3.62, 5.15) [0.016]	Not done	2.17 (1.67, 2.76) [0.000]	0.3 (2.41, 3.9) [0.000]	4.5 (3.77, 5.33) [0.011]	2.1 (1.61, 2.69) [0.000]	7.4 (6.46, 8.44) [0.026]	9.83 (8.74, 11.02) [–]

*P-value for test of mean differences between intervention and control arms

Intervention: Indoor residual spraying (IRS), Long lasting insecticide impregnated bed-net (LLIN), Local nets impregnated with slow release insecticide tablets K-O TAB 1-2-3 (KOTAB), Possible sand fly breeding places around house sprayed with chlorpyrifos (OUT), control = no intervention provided

### Efficacy of the intervention and bioavailability of the insecticide on treated surfaces

The efficacy of the interventions was measured through the reduction of *P*. *argentipes* sand fly densities in intervention HHs compared to the control HHs. The bioavailability of insecticides on treated surfaces (indicating how long the insecticide was capable of killing insect vectors) was determined using bioassay tests.

#### Intervention effect on sand fly density

(i) Two consecutive cycles of IRS were effective in significantly reducing sand fly vector densities for more than 12 months (January 2013 to March 2014). The reduction of sand fly vector densities was 14% to 80% at the different points of measurement and the rate ratio (RR) of *P*. *argentipes* sand fly counts after and before the intervention was 0.86 and 0.20 up to a 15 months period; the differences were statistically significant by the adjusted model. (ii) The LLIN was effective from the second follow-up onwards throughout the study period (Table 4). The adjusted model for LLIN showed that the vector density reduction was 9% to 78% and the rate ratio was between 0.91 and 0.32 for two years. (iii) In the KOTAB arm, a significant sand fly density reduction was observed only at the third (RR = 0.90, p = 0.38), sixth (RR = 0.33, p = 0.050), seventh (RR = 0.56, p = 0.003) and eighth (RR = 0.89, p = 0.010) follow-up measurements. (iv) In the OUT arm, statistically insignificant *P*. *argentipes* sand fly density reductions and RRs were observed (Table 4).

**Table 4:** Efficacy of individual insecticides and their combinations used in different intervention arms after adjusting for confounders by longitudinal regression analysis of total number of *P*. *argentipes* in Fulbaria, Mymensingh, Bangladesh.

Measurement/Model	Parameter	Incidence rate ratio, IRR (P-value) [95% CI]								
		IRS	LLIN	KOTAB	OUT	IRS+LLIN	IRS+KOTAB	IRS+OUT	LLIN+OUT	KOTAB+OUT
**1st follow-up**										
Simple model	*Crude Intervention effect*	0.34(0.014)[0.14–0.80]	0.65(0.188)[0.34–1.24]	0.41(0.055)[0.16–1.02]	0.84(0.560)[0.46–1.52]	–*	0.21(0.031) [0.05–0.87]	0.15(0.020) [0.03–0.74]	0.34(0.038)[0.12–0.94]	0.68(0.294)[0.33–1.39]
Full model	*Adjusted Intervention effect*	0.37(0.036) [0.15–0.94]	0.69(0.265)[0.36–1.33]	0.40(0.060)[0.16–1.04]	0.88(0.689)[0.47–1.65]	**–** *	0.22(0.031)[0.05–0.87]	0.15(0.023)[0.03–0.77]	0.36(0.056)[0.13–1.03]	0.72(0.385)[0.34–1.52]
**2nd follow-up**										
Simple model	*Crude Intervention effect*	0.35(0.000)[0.20–0.62]	0.78(0.054)[0.61–1.00]	0.84(0.172)[0.66–1.08]	1.02(0.799)[0.87–1.20]	0.19(0.001) [0.07–0.52]	0.84(0.104) [0.68–1.04]	0.12(0.001) [0.03–0.43]	0.42(0.002)[0.24–0.73]	0.81(0.098)[0.64–1.04]
Full model	*Adjusted Intervention effect*	0.37(0.002) [0.20–0.71]	0.78(0.047)[0.61–1.00]	0.83(0.154)[0.65–1.07]	0.99(0.960)[0.85–1.17]	0.18(0.002)[0.06–0.55]	0.84(0.178)[0.65–1.08]	0.12(0.001)[0.03–0.44]	0.41(0.002)[0.23–0.72]	0.80(0.090)[0.61–1.04]
**3rd follow-up**										
Simple model	*Crude Intervention effect*	0.74(0.000)[0.63–0.86]	0.90(0.018)[0.82–0.98]	0.90(0.038)[0.82–0.99]	0.99(0.759)[0.93–1.05]	0.40(0.000) [0.25–0.64]	0.69(0.000) [0.58–0.84]	0.56(0.000) [0.41–0.76]	0.67(0.001)[0.53–0.84]	0.96(0.183)[0.90–1.02]
Full model	*Adjusted Intervention effect*	0.74(0.000) [0.63–0.86]	0.86(0.006)[0.78–0.96]	0.91(0.034)[0.83–1.00]	0.95(0.114)[0.89–1.01]	0.39(0.000)[0.24–0.63]	0.70(0.000)[0.59–0.84]	0.54(0.000)[0.39–0.74]	0.60(0.000)[0.46–0.79]	0.93(0.023)[0.88–0.99]
**4th follow-up**										
Simple model	*Crude Intervention effect*	0.86(0.003) [0.77–0.95]	0.91(0.035)[0.84–0.99]	0.95(0.224)[0.87–1.03]	1.01(0.854)[0.95–1.07]	0.64(0.001) [0.49–0.84]	0.64(0.001) [0.50–0.83]	0.67(0.001) [0.52–0.85]	0.75(0.001) [0.63–0.90]	0.98(0.523)[0.93–1.04]
Full model	*Adjusted Intervention effect*	0.87(0.008)[0.80–1.00]	0.91(0.035)[0.83–1.00]	0.96(0.223)[0.90–1.23]	1.01(0.801)[0.95–1.06]	0.65(0.002)[0.50–0.85]	0.65(0.001)[0.50–0.84]	0.66(0.001)[0.51–0.85]	0.74(0.002) [0.61–0.89]	0.99(0.545)[0.94–1.03]
**5th follow-up**										
Simple model	*Crude Intervention effect*	0.31(0.000)[0.17–0.58]	0.45(0.002)[0.27–0.75]	0.85(0.130)[0.69–1.05]	0.97(0.671)[0.83–1.13]	0.27(0.001) [0.12–0.60]	0.15(0.001) [0.05–0.45]	0.21(0.001) [0.08–0.55]	0.40(0.002)[0.22–0.73]	0.87(0.170)[0.71–1.06]
Full model	*Adjusted Intervention effect*	0.34(0.002)[0.17–0.68]	0.44(0.002)[0.26–0.74]	0.84(0.159)[0.67–1.07]	0.96(0.574)[0.82–1.11]	0.26(0.001)[0.12–0.60]	0.15(0.000)[0.05–0.42]	0.21(0.002)[0.08–0.56]	0.39(0.003)[0.21–0.72]	0.88(0.179)[0.73–1.06]
**6th follow-up**										
Simple model	*Crude Intervention effect*	0.20(0.014)[0.05–0.72]	0.32(0.058)[0.10–1.04]	0.33(0.050)[0.11–0.10]	0.52(0.136)[0.22–1.23]	0.14(0.034) [0.02–0.86]	0.31(0.040) [0.10–0.95]	0.14(0.020) [0.03–0.73]	0.14(0.020)[0.03–0.73]	0.26(0.038)[0.07–0.93]
Full model	*Adjusted Intervention effect*	0.25(0.052)[0.06–1.01]	0.22(0.036)[0.05–0.91]	0.27(0.045)[0.08–0.97]	0.58(0.245)[0.23–1.46]	0.15(0.055)[0.02–1.04]	0.31(0.048)[0.10–1.00]	0.15(0.038)[0.02–0.90]	0.14(0.032)[0.02–0.85]	0.28(0.061)[0.07–1.06]
**7th follow-up**										
Simple model	*Crude Intervention effect*	0.54(0.000)[0.39–0.75]	0.58(0.002)[0.41–0.82]	0.56(0.003)[0.38–0.82]	0.71(0.009)[0.55–0.92]	0.35(0.000) [0.20–0.61]	0.14(0.000) [0.05–0.40]	0.44(0.001) [0.28–0.71]	0.28(0.001)[0.13–0.58]	0.54(0.002)[0.37–0.80]
Full model	*Adjusted Intervention effect*	0.56(0.001)[0.40–0.80]	0.57(0.002)[0.40–0.81]	0.56(0.003)[0.38–0.83]	0.69(0.010)[0.52–0.92]	0.35(0.000)[0.20–0.63]	0.14(0.000)[0.05–0.40]	0.43(0.001)[0.26–0.71]	0.27(0.001)[0.12–0.58]	0.54(0.003)[0.37–0.80]
**8th follow-up**										
Simple model	*Crude Intervention effect*	Not done	0.90(0.008)[0.84–0.97]	0.89(0.010)[0.81–0.97]	Not done	0.72(0.000) [0.60–0.85]	0.58(0.000) [0.44–0.75]	0.76(0.000) [0.66–0.88]	0.71(0.000)[0.60–0.85]	0.96(0.134)[0.90–1.01]
Full model	*Adjusted Intervention effect*	Not done	0.87(0.008)[0.81–0.97]	0.87(0.005)[0.80–0.96]	Not done	0.70(0.000)[0.57–0.85]	0.58(0.000)[0.45–0.74]	0.74(0.001)[0.62–0.89]	0.64(0.000)[0.50–0.81]	0.95(0.040)[0.90–1.00]
**9th follow-up**										
Simple model	*Crude Intervention effect*	Not done	0.82(0.005)[0.71–0.94]	0.93(0.124)[0.85–1.02]	Not done	0.77(0.002) [0.65–0.91]	0.82(0.004) [0.72–0.93]	0.92(0.061) [0.84–1.00]	0.75(0.002)[0.62–0.90]	0.98(0.570)[0.92–1.05]
Full model	*Adjusted Intervention effect*	Not done	0.81(0.006)[0.70–0.94]	0.94(0.125)[0.86–1.02]	Not done	0.77(0.003)[0.65–0.92]	0.84(0.006)[0.74–0.95]	0.92(0.058)[0.84–1.00]	0.74(0.002)[0.61–0.90]	0.99(0.628)[0.94–1.04]

* IRR was not estimated trough model due to computational limitation (convergence not achieved) related to no variation in sand fly count in intervention clusters at 1^st^ follow-up

Intervention: Indoor residual spraying (IRS), Long lasting insecticide impregnated bed-net (LLIN), Local nets impregnated with slow release insecticide tablets K-O TAB 1-2-3 (KOTAB), Possible sand fly breeding places around house sprayed with chlorpyrifos (OUT)

**Fig 4:**
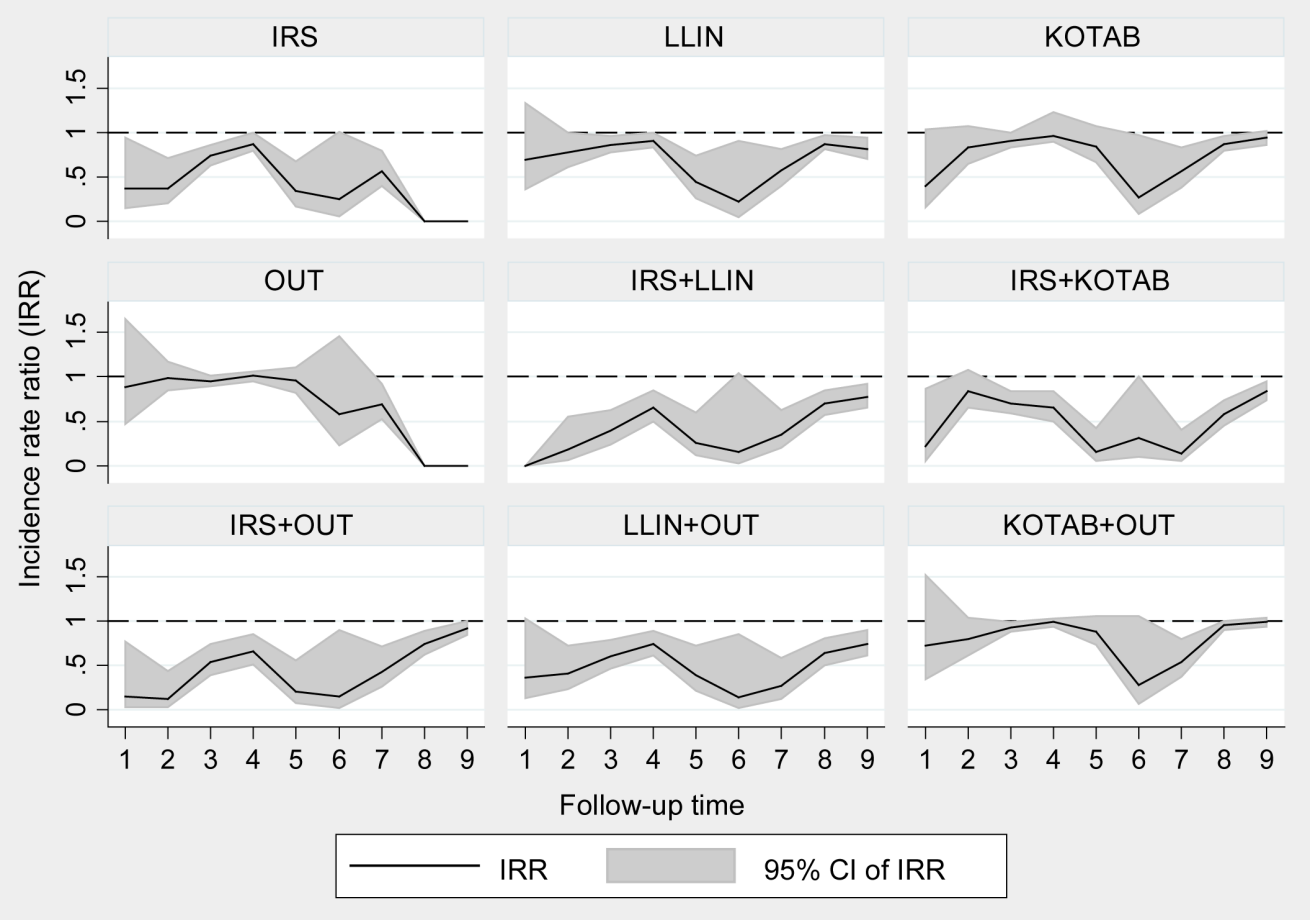
Benefit of interventions attributed by reduction of *P*. *argentipes* sand fly density at household level.

In the other intervention arms, different methods were combined. (v) The combination of IRS with LLIN (IRS+LLIN) or (vi) with KOTAB (IRS+KOTAB) and (vii) with outdoor spray (IRS+OUT) showed statistically significant *P*. *argentipes* reductions throughout the study period except at the second follow-up (p = 0.178 for adjusted model) for IRS+KOTAB (Table 4). The adjusted model showed that the effects of IRS+LLIN, IRS+KOTAB, and IRS+OUT varied respectively from 23.0% density reduction (RR = 0.77, p = 0.003) to 85.0% (RR = 0.15, p = 0.055), 16.0% (RR = 0.84, p = 0.178) to 86.0% (RR = 0.14, p = <0.0001) and 8.0% (RR = 0.92, p = 0.058) to 88.0% (RR = 0.12, p = 0.001). Likewise, the combination (viii) of LLIN with outdoor spray (LLIN+OUT) was found to be effective throughout the study period at a highly significance level. The reduction of *P*. *argentipes* sand fly density attributed to LLIN+OUT was 26.0% (RR = 0.74, p = 0.002) to 86.0% (RR = 0.14, p = 0.032). However, the combination of (ix) outdoor spraying with KOTAB (OUT+KOTAB) had no statistically significant effect on density reduction of *P*. *argentipes* sand fly counts in most of the follow-up points (Table 4). All interventions reduced vector density and were effective (except OUT alone) throughout the study period (Fig 4).

### Sand fly mortality (bioassay) on treated surfaces

**Fig 5:**
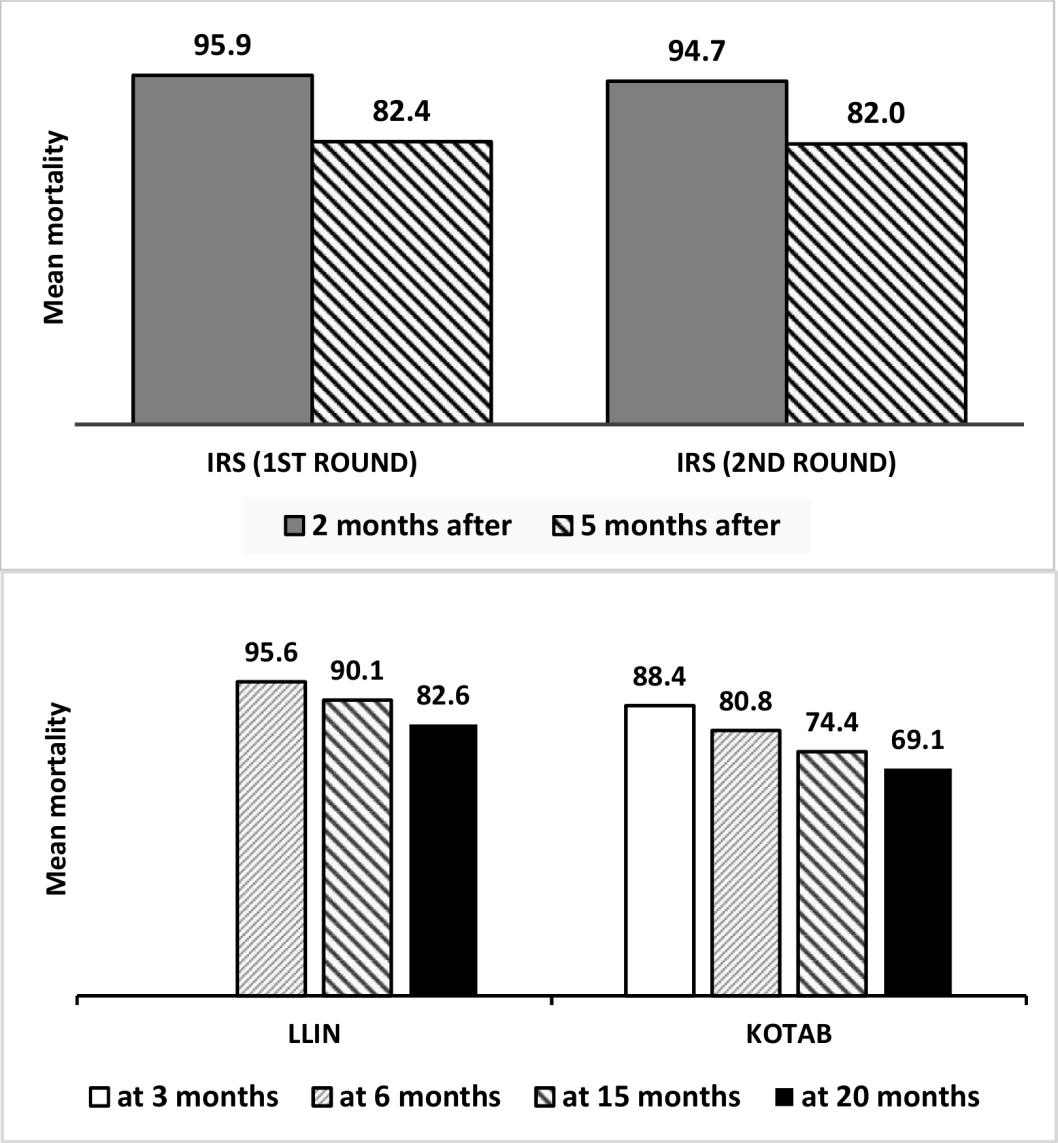
Abbot-corrected *P*. *argentipes* sand fly mortality by intervention at follow up periods.

The Abbot corrected sand fly mortality at 24 hours of exposure on treated surfaces was as follows: in both cycles of IRS, the mortality was above 80% (which is the threshold level) even after 5 months following spraying (Fig 5). The mortality for LLIN was 82.59% at 20 months of use. The mortality on K-O TAB 1-2-3 impregnated nets dropped from 88.37% at 3 months to 69.12% at 20 months after use.

## Discussion

Our cluster randomized controlled trials on VL vector interventions are to our knowledge the largest ever conducted in the South-East Asia Region. In the present study a large number of sand flies was captured of which 45.9% were other than *P*. *argentipes* species which is three times higher than in a previous study conducted in the same sub-district [10]. We have tested four individual types of interventions and five combinations against VL vectors. In the present study, we used alpha cypermethrin 5WP for IRS as it is less expensive than deltamethrin and as efficacious as other pyrethroids [10,20]. IRS is however challenging in terms of operational complexity and cost and it is difficult to maintain a uniform quality of spraying. It was observed in India and Nepal that when IRS was applied by the research team under well controlled conditions, it was found to be very effective against VL vectors but when it was delivered by the national programme the efficacy dropped significantly [11].

At the current stage of the VL elimination initiative in the Indian subcontinent, Nepal has reached the target of less than one case per 10,000 population, Bangladesh has only a few sub-districts (upazilas) above this threshold and India has reached the goal in many areas but is still facing elevated VL endemicity in a number of districts [30]. In sub-districts where the final push towards elimination is still required, IRS alone or in combination with other measures are still needed. In sub-districts where VL/PKDL cases appear sporadically and case numbers are below the thresholds, new ways of active case detection (to reduce the transmission) and vector control (to prevent transmission) are required.

Regarding vector management in the post-elimination phase, recent studies have shown the potential of different vector control tools, including insecticide treated durable wall lining (DWL), commercially impregnated long lasting insecticidal nets (LLIN), slow release insecticides (K-O TAB 1-2-3,) treatment of existing bed nets (ITNs), as well as insecticidal paint (Inesfly company, Valencia, Spain). Prospects and limitations of these products include the following:

- DWL has a long lasting effect (tested for 12 months) [31], can be reduced in size covering only the lower parts of the wall without losing efficacy [32]. The product is however costly and the application on the walls is not easy [31,33].
- Commercial LLINs have shown contradictory results regarding their efficacy. In an older study in India and Nepal, LLIN did not reduce infection rates [34], but in subsequent studies in Bangladesh and then again in India and Nepal, LLIN showed a significant reduction of VL vectors [10,20]. Their general use will depend on external funding.
- ITNs showed a significant reduction of VL vectors in various studies [12,31], the duration of the effect was up to 12 to 18 months.

Our study contributes important information to what is known already. (i) The combination of different approaches leads to better results than single approaches in reducing the vector population. (ii) The combination of a chemical intervention in breeding and larval-development sites in and around rural houses together with measures against adult vectors using LLINs was particularly successful in significantly reducing vector populations for at least 22 months. Furthermore, this measure can be used in remote areas where sporadic cases appear as it lends itself to community actions. The use of ITNs would be even more feasible as it is independent of LLIN donations. Local bed nets to prevent mosquito bites are common in rural Bangladeshi communities and over 90% of HHs have nets [31,35]. Slow release insecticide impregnated bed nets might be a good alternative to prevent sand fly bites but the effect on the vector population was shorter and less marked compared to LLINs.

It will be a remarkable innovation if applications of insecticides in breeding places of sand flies around houses in endemic communities are able to reduce the vector density. In the current study, we tested chlorpyrifos 20EC to control immature stages of sand flies because this insecticide has no/very limited side effects on the environment or on human health [36]. The result following the first round of chlorpyrifos spraying was not promising but after the second round of spraying, it was effective in reducing the vector densities. This effect was considerably enhanced when combined with the treatment of bed nets.

In conclusion: The combination of LLIN and OUT (outdoor spraying of vector breeding sites) was the most efficacious measure among the different tools tested. This combination measure could play an important role during the maintenance phase of the VL elimination programme to maintain a low vector density particularly in remote areas where the community can take care of the measure. The relationship between vector density (males plus females) and leishmaniasis incidence should be investigated, and this will require estimates of the Entomological Inoculation Rate.

## Supporting information

S1 Table Male and female comparison of *P*. *argentipes* sand fly and their mean. (DOCX)
