# Supplementary material for: Control of Phlebotomus argentipes (Diptera: Psychodidae) sand fly in Bangladesh: A cluster randomized controlled trial
Source: PLoS Negl Trop Dis. 2017 Sep 5;11(9):e0005890. doi: 10.1371/journal.pntd.0005890 (PMC5600390; doi:10.1371/journal.pntd.0005890)
Supplement: S1 Table — (DOCX) [file pntd.0005890.s001.docx]

Table: Male and female comparison of *P. argentipes* sand fly and their mean

| **Study arm** | **Control** | **IRS** | **LLIN** | **KOTAB** | **OUT** | **IRS+LLIN** | **IRS+KOTAB** | **IRS+OUT** | **LLIN+OUT** | **KOTAB+OUT** |
| --- | --- | --- | --- | --- | --- | --- | --- | --- | --- | --- |
| **Measurement point** | **p value [mean – male,female]** | | | | | | | | | |
| Baseline (October and November 2012) | 0.565  [4.9,5.667] | 0.061  [5.333,6.2] | 0.519  [4.567,4.233] | 0.992  [3.4,3.9] | 0.054  [5.0,3.7] | 0.626  [4.633,4.0] | 0.024  [4.733,6.1] | 0.013  [4.933,3.767] | 0.365  [4.833,5.133] | 0.185  [5.133,4.233] |
| First follow up (February 2013) | 0.109  [0.567,0.167] | 0.564  [0.067,0.1] | 0.958  [0.2,0.167] | 0.083  [0.167,0.033] | 0.690  [0.3,0.233] | -  - | 0.317  [0.067,0.033] | 0.157  [0.067,0.0] | 0.025  [0.167,0.0] | 0.675  [0.233,0.167] |
| Second follow up(April 2013) | 0.235  [1.8,1.333] | 0.366  [0.233,0.133] | 0.632  [0.867,0.533] | 0.365  [0.967,0.633] | 0.024  [1.9,1.267] | 0.180  [0.133,0.033] | 0.489  [0.8,0.967] | 0.564  [0.067,0.033] | 0.965  [0.167,0.3] | 0.951  [0.667,0.9] |
| Third follow up (May 2013) | 0.244  [7.067,5.033] | 0.160  [0.9,1.267] | 1.000  [2.233,2.2] | 0.281  [1.5,2.6] | 0.703  [4.867,3.933] | 0.119  [0.333,0.233] | 0.622  [0.8670.933] | 0.470  [0.567,0.467] | 0.512  [0.8,0.8] | 0.213  [4.1,3.0] |
| Forth follow up (July 2013) | 0.012  [6.233,4.6] | 0.670  [1.867,1.9] | 0.035  [2.7,1.867] | 0.344  [3.067,2.067] | 0.128  [5.067,4.333] | 0.639  [0.667,0.7] | 0.267  [0.6,0.833] | 0.380  [0.633,0.867] | 0.940  [1.067,1.1] | 0.241  [4.467,3.5] |
| Fifth follow up (November 2013) | 0.761  [1.9,1.9] | 0.206  [0.1, 0.233] | 0.246  [0.167,0.367] | 0.558  [0.933,0.9] | 0.597  [1.6,1.433] | 0.046  [0.2,0.067] | 1.000  [0.067,0.067] | 0.202  [0.133,0.067] | 0.039  [0.1,0.367] | 0.634  [1.067,1.067] |
| Sixth follow up (February 2014) | 0.033  [0.567,0.267] | 0.317  [0.067,0.33] | 0.655  [0.1,0.067] | 0.025  [0.1667,0.0] | 0.309  [0.233,0.067] | 1.000  [0.033,0.033] | 0.655  [0.067,0.1] | 0.564  [0.033,0.067] | 0.317  [0.067,0.0] | 0.551  [0.033,0.1] |
| Seventh follow up (March 2014) | 0.908  [2.467,2.4] | 0.838  [0.4,0.0.433] | 0.338  [0.4,0.5] | 0.989  [0.3667,0.4333] | 0.752  [0.767,0.6] | 0.788  [0.2,0.2] | 0.551  [0.033,0.1] | 0.929  [0.233,0.333] | 0.083  [0.2,0.1] | 0.496  [0.3,0.5] |
| Eighth follow up (June 2014) | 0.106  [12.1,8.167] | N/A | 0.884  [2.8,2.667] | 0.471  [2.3,2.133] | N/A | 0.074  [1.433,0.6] | 0.250  [0.767,0.467] | 0.407  [1.4,1.1] | 0.927  [1.167,0.933] | 0.924  [5.266,4.0] |
| Ninth follow up (October 2014) | 0.772  [4.8,5.033] | N/A | 0.343  [1.3,1.367] | 0.664  [2.0,2.333] | N/A | 0.889  [1.067,1.1] | 0.944  [1.467,1.533] | 0.178  [1.967,2.533] | 0.129  [0.9,1.2] | 0.464  [3.8,3.6] |
